# Mathematical and Dynamic Modeling of the Anatomical Localization of the Insula in the Brain

**DOI:** 10.1007/s12021-025-09727-4

**Published:** 2025-04-23

**Authors:** Eren Ogut

**Affiliations:** Faculty of Medicine, Department of Anatomy, Istanbul Medeniyet University, Istanbul, 34700 Türkiye

**Keywords:** Insula, Mathematical modeling, Functional connectivity, Anatomical localization, MNI coordinates

## Abstract

The insula, a deeply situated cortical structure beneath the Sylvian sulcus, plays a critical role in sensory integration, emotion regulation, and cognitive control in the brain. Although several studies have described its anatomical and functional characteristics, mathematical models that quantitatively represent the insula’s complex structure and connectivity are lacking. This study aimed to develop a mathematical model to represent the anatomical localization and functional organization of the insula, drawing on current neuroimaging findings and established anatomical data. A three-dimensional (3D) ellipsoid model was constructed to mathematically represent the anatomical boundaries of the insula using Montreal Neurological Institute (MNI) coordinate data. This geometric model adapts the ellipsoid equation to reflect the spatial configuration of the insula and is primarily based on cytoarchitectonic mapping and anatomical literature. Relevant findings from prior imaging research, particularly those reporting microstructural variations across insular subdivisions, were reviewed and conceptually integrated to guide the model’s structural assumptions and interpretation of potential applications. The ellipsoid-based 3D model accurately represented the anatomical dimensions and spatial localization of the right insula, centered at the MNI coordinates (40, 5, 5 mm), and matched well with the known volumetric data. Functional regions (face, hand, and foot) were successfully plotted within the model, and statistical analysis confirmed significant differences along the anteroposterior and superoinferior axes (*p* < 0.01 and *p* < 0.05, respectively). Dynamic simulations revealed oscillatory patterns of excitatory and inhibitory neural activity, consistent with established insular neurophysiology. Additionally, connectivity modeling demonstrated strong bidirectional interactions between the insula and key regions, such as the prefrontal cortex and anterior cingulate cortex (ACC), reflecting its integrative role in brain networks. This study presents a scientifically validated mathematical model that captures the anatomical structure, functional subdivisions, and dynamic connectivity patterns of the insula. By integrating anatomical data with computational simulations, this model provides a foundation for future research in neuroimaging, functional mapping, and clinical applications involving insula-related disorders.

## Introduction

**Fig. 1:**
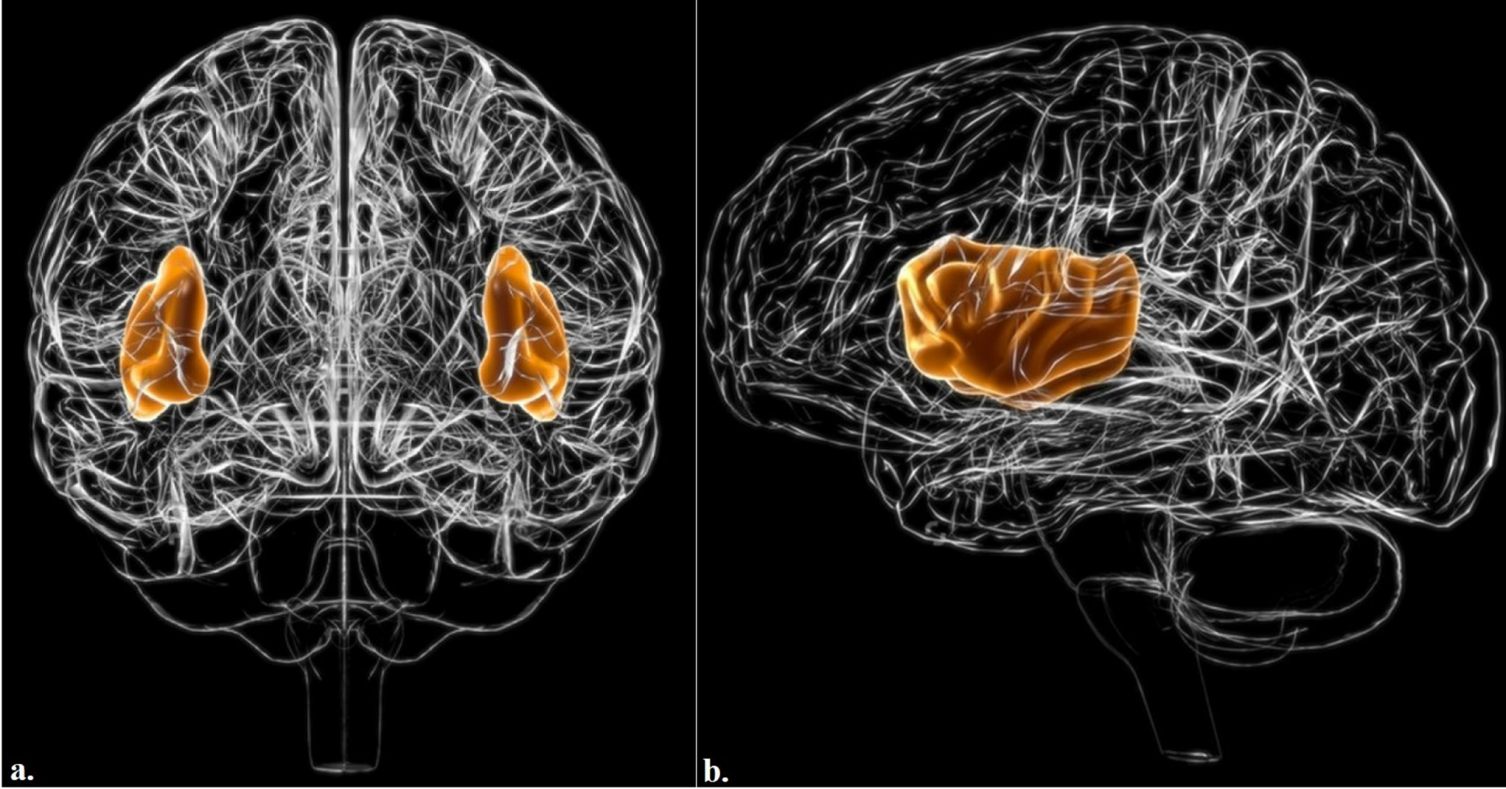
3D reconstruction of insula. Anterior (**a**) and lateral (**b)** views are presented. The frontal, parietal, and temporal lobes cover the insula, which forms the floor of the lateral sulcus. The insula becomes visible when the frontal, parietal, and temporal opercula are removed

The insula is a deep structure of the brain located laterally and hidden beneath the Sylvian sulcus (Ghaziri, 2018). The insula is the only cortical part of the brain that is not visible on the surface of the hemisphere, as it is entirely covered by the frontoparietal and temporal opercula (Guenot et al., 2004; Mesulam, 2000) (Fig. 1). The insula has a triangular shape and is separated from the opercula by the anterior, superior, and inferior peri-insular sulci (Guenot et al., 2004). It is often regarded as the fifth lobe of the brain because of its strategic location and multimodal role (Ghaziri, 2018). The insula is morphologically divided into two parts by the central insular sulcus. The anterior part has three short gyri, and the posterior part has two long gyri (Guenot et al., 2004; Türe et al., 1999; Uddin et al., 2017). Its unique position and complex anatomy contribute to its involvement in a wide range of functions, including sensory processing, affective processing, and high-level cognition (Uddin et al., 2017; Cauda et al., 2011). Its anatomical localization has been studied using several methods, including cytoarchitectonic mapping, Diffusion Magnetic Resonance Imaging (MRI), functional MRI, geometric MRI-based modeling and other neuroimaging techniques (Table 1). Quantitative modeling has revealed significant differences in microstructure across insular subdivisions, serving distinct cognitive and affective functions (Menon, 2020). This microstructural organization was mirrored in functionally interconnected circuits within the ACC, which anchors the salience network. Insular microstructural features are also linked to behavior and predict individual differences in cognitive control ability. Interestingly, the insula exhibits a gradual transition in connectivity patterns, as revealed by probabilistic white matter tractography and Laplacian eigenmaps (Cerliani et al., 2011). Despite these advances, the development of mathematical models for the anatomical and functional representation of the insula remains limited to date. The current lack of such models cannot be solely attributed to the absence of prior research; rather, it reflects a gap in integrating mathematical modelling into neuroanatomical studies of the insula. Mathematical and computational models have proven to be highly effective in other brain regions. For example, the hippocampus has been modeled using neural mass models to simulate epileptic activity and memory-encoding processes (Wendling et al., 2002; Cutsuridis et al., 2010). Similarly, the cerebral cortex has been studied using graph theoretical approaches and dynamic causal modeling to understand network-level interactions and disease progression in conditions such as Alzheimer’s disease (Friston et al., 2003; Stam, 2014). Therefore, the application of mathematical and dynamic modeling approaches to the insula is not only timely but also essential for advancing our understanding of its microstructure, connectivity, and role in higher-order functions. The establishment of certain models can also facilitate the development of diagnostic tools and therapeutic interventions for insula-related disorders, including anxiety, depression, and epilepsy. This study aims to fill the gap in neuroanatomical research by developing a mathematical and dynamic model of the insula that accurately represents its anatomical localization, functional subdivisions, and network-level connectivity, thereby contributing to the understanding of its role in health and diseases.

**Table 1:** Summary of studies on anatomical localization of the Insula and their relevance to mathematical modeling

Study	Model Type	Anatomical Localization Method	Parameters Calculated	Contribution to Mathematical/Dynamic Modeling
Ghaziri et al. (2018)	Structural Connectivity Map	Diffusion MRI	Subregional connectivity in the insula	Informs network-based models; defines connectivity matrix for simulations.
Cauda et al. (2011)	Functional Connectivity Map	Functional Neuroimaging /fMRI	Resting-state network correlations	Supports dynamic models of network interactions, salience processing.
Bauernfeind et al. (2013)	Comparative Morphology	Structural MRI + Morphometrics	Insular subregion volumes in primates	Basis for evolutionary modeling; defines morphometric constraints.
Kurth et al. (2009)	Probabilistic Mapping	Cytoarchitectonic Mapping	Posterior insula cytoarchitecture and maps	Delivers high-resolution anatomical data for precise structural modeling.
Zhang et al. (2024)	Multimodal Functional Model	Combined (Diffusion MRI + fMRI)	Sensory, emotional, and cognitive integration	Combines structure-function data for dynamic simulations of integrative function.
Türe et al. (1999)	Topographic Atlas	Topographic Anatomy (Dissection)	Anatomical subdivisions of the insula	Provides spatial data for geometric modeling; supports ellipsoid model input.
Menon, (2020)	Microstructural Gradient Model	Diffusion MRI + Functional MRI	Insular microstructure and macrofunctional circuits	Identifies microstructural gradients for parameterizing dynamic models of cognition.

**Abbreviations: fMRI**: Functional Magnetic Resonance Imaging, **MRI**: Magnetic Resonance Imaging

### Function

The insula is a complex and deeply situated brain region with diverse functions that are tightly linked to its anatomical structure and extensive connectivity (Zhang et al., 2024). Owing to its unique position and widespread afferent and efferent connections, the insula serves as a critical integrative hub within functional brain networks (Türe et al., 1999; Uddin et al., 2017; Cauda et al., 2011; Zhang et al., 2024; Ogut et al., 2023; Rodgers et al., 2008). Anatomically, the brain is divided into anterior and posterior regions, each with distinct functional roles. The posterior insula is primarily involved in processing auditory and somatosensory stimuli, with somatotopically organized fields and intermediate zones that can integrate multisensory inputs (Rodgers et al., 2008). In contrast, the anterior insula is engaged in higher-order processes, such as emotional awareness, interoception, and cognitive control (Zhang et al., 2024; Tisserand, 2023). Several studies have elucidated the functional heterogeneity, including its involvement in the non-motor aspects of neurological disorders, such as Parkinson’s disease (Güzelad et al., 2021), and its integration into critical brain networks, such as the salience network (Criaud et al., 2016). Cytoarchitectonic investigations have identified three distinct microstructural areas in the posterior insula, laying the groundwork for understanding its functional segregation at the cellular level (Kurth et al., 2009). However, these insights are primarily based on qualitative analyses and lack explicit mathematical modeling of the insula (Criaud et al., 2016; Pavuluri et al., 2015). It serves as a receiver and interpreter of emotions in the context of cognitive and sensory-motor information, making it crucial for interoception, emotion processing, and decision-making (Zhang et al., 2024; Pavuluri et al., 2015; Ogut et al., 2020; Fermin et al., 2022). The insula’s function progresses from receiving and interpreting sensorimotor sensations in the posterior region to the subjective perception of emotions in the anterior region (Pavuluri et al., 2015; Fermin et al., 2022). This hierarchical organization is reflected in the Insula Hierarchical Modular Adaptive Interoception Control model, which suggests that insular modules form higher-order interoceptive representations that support control processes (Fermin et al., 2022). The anatomical structure and connectivity of the insula are linked to its diverse functions, making it a crucial interface between sensation, emotion, and cognition. Its importance in various brain processes and potential as a biomarker for treatment selection has been previously reported (Zhang et al., 2024; Pavuluri et al., 2015). Existing research predominantly discusses the anatomical, functional, and clinical aspects of the insula in descriptive terms without introducing mathematical equations or models to quantify its structure or behavior. For instance, studies by Menon et al. and Uddin et al. explored the insular microstructure and its involvement in the salience network (Uddin et al., 2017; Menon, 2020), while Sheffield et al. examined volumetric and morphological variations in psychosis-spectrum disorders (Sheffield, 2021). These contributions, while highly valuable, do not constitute formal mathematical modeling but offer quantitative neuroimaging data that can inform model construction. To date, there is limited literature on mathematical or dynamic modeling directly applied to the insular cortex. However, approaches such as finite element modeling and neural network simulations, although not specifically designed for the insula, illustrate how anatomical data can be incorporated into computational models to simulate brain tissue behavior and neural dynamics (Fermin et al., 2022; Chen, 2010; Brunel, 2000). These approaches serve as templates for developing mathematical representations of the insula; therefore, this study aims to address this gap by developing a mathematical model for the insula, grounded in existing anatomical and neuroimaging data. We propose an ellipsoid-based geometric model to represent the spatial configuration of the insula, informed by MNI coordinate systems and validated through literature-reported microstructural and functional patterns.

### Coordinate System and Mathematical Localization

The Talairach and MNI coordinate systems are widely used for mathematical localization of brain regions in neuroimaging studies. These systems provide a standardized framework for reporting and comparing brain locations across different studies and participants (Brett, 2001; Chau & McIntosh, 2005). The Talairach coordinate system, which is based on Talairach and Tournoux’s 1988 atlas, has become a standard reference for reporting brain locations in literature. However, many functional imaging studies now match their data to the MNI template, which is based on the International Consortium of Brain Mapping (Brett, 2001; Chau & McIntosh, 2005). This has led to some challenges, as the brains in the Talairach atlas and MNI template differ significantly in terms of shape and size (Brett, 2001). To address these discrepancies, researchers have developed several methods for converting coordinates between the two systems. These include the Talairach Method of Piecewise Linear Scaling and template-matching approaches using linear transformations (Chau & McIntosh, 2005). Additionally, automated nonlinear transformations have been created to convert the coordinates from one brain to a corresponding point in the other (Brett, 2001; Lacadie et al., 2008). However, it is important to note that there can be significant discrepancies in certain brain regions when converting between these systems, particularly in the inferior, superior frontal, and occipital regions (Chau & McIntosh, 2005).

These systems define:

- **x-axis**: mediolateral plane,
- **y-axis**: anterior-posterior plane, and.
- **z-axis**: superior-inferior plane.

The average MNI coordinates for the insula were as follows:

- **Right insula**: x = 40, y = 5, z = 5 mm.
- **Left insula**: x = − 40, y = 5, z = 5 mm.

**Fig. 2:**
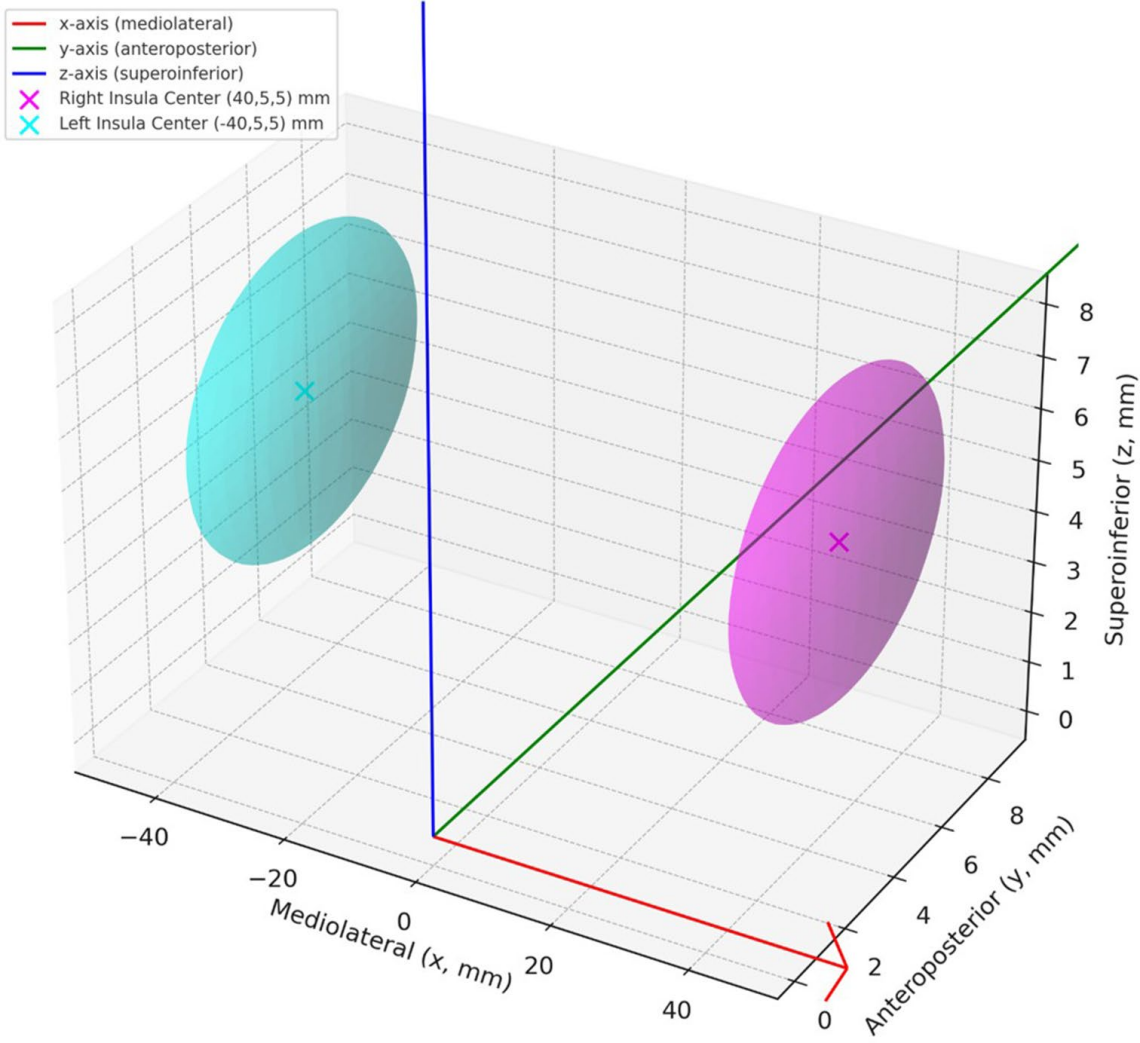
3D diagram illustrating the MNI coordinate system and the central anatomical locations of the right and left insula. The red, green, and blue arrows represent the **x** (mediolateral), **y** (anteroposterior), and **z** (superoinferior) axes, respectively. The magenta and cyan spheres indicate the locations and spatial dimensions of the right and left insula, centered at (40, 5, 5) mm and (–40, 5, 5) mm, respectively

In our study, the MNI coordinates of the insula (right insula: x = 40, y = 5, z = 5 mm; left insula: x = − 40, y = 5, z = 5 mm) were mathematically derived to serve as reference points for the geometric modeling of the insula within the standard brain space. These coordinates were not determined through simulation or empirical stimulation-based measurements but were calculated to represent the anatomical location of the insula in the standard MNI space for constructing a geometric ellipsoid model. The selection of these values was informed by general anatomical knowledge and commonly reported insular positions in neuroimaging atlases rather than task-based or function-specific data (Fig. 2).

Multiple studies have demonstrated variability in the reported MNI coordinates depending on the specific region targeted and the nature of the task or stimulation involved. For example, Brooks et al. provided MNI coordinates reflecting somatosensory activation in the insula (face: −40, − 16, 11) (Brooks et al., 2005), while Mazzola et al. reported vestibular, gustatory, and olfactory responses in different insular subregions. These findings highlight the functional complexity and spatial variability of insular activity (Mazzola et al., 2014). Given this variability, the coordinates used in our model were intended to provide a simplified geometric center for mathematical representation and not to reflect any specific functional domain or task-related activation site. Task-based studies yield more posterior or superior coordinate values, as reported by (Brooks et al., 2005), and the y and z coordinates provided in the question do not accurately represent the location of insular activity. However, our approach does not involve such task-specific determinations.

## Materials and Methods

### Mathematical Modeling of the Insula

A 3D ellipsoid model was constructed to mathematically represent the anatomical localization and spatial dimensions of the right insula within the MNI coordinate system(Mitteroecker & Gunz, 2009).

The ellipsoid equation used in this study follows the standard mathematical form as follows:

$${\left( {x - {x_0}} \right)^2}/{a^2} + {\left( {y - {y_0}} \right)^2}/{b^2} + {\left( {z - {z_0}} \right)^2}/{c^2} = 1$$

where (x_0_,y_0_,z_0_) are the *center coordinates* of the ellipsoid model, corresponding to the mediolateral, anteroposterior, and superoinferior dimensions, respectively. In our study, this ellipsoid equation was constructed to approximate the anatomical position and spatial dimensions of the right insula using the standard MNI coordinate space. The rationale for the selected parameters was based on the average insular dimensions reported in anatomical and neuroimaging studies. The *center coordinates* were set at (x_0_ = 40 mm, y_0_ = 5 mm, z_0_ = 5 mm), representing the general central location of the right insula within the MNI coordinate system. The semi-axes were determined from volumetric data: a = 12 mm (mediolateral extent), b = 8 mm (anteroposterior extent), c = 6 mm (superoinferior extent). These values correspond to the known anatomical ranges for insular dimensions obtained from neuroimaging volumetric studies (Faillenot et al., 2017; Makris et al., 2006). The resulting ellipsoid equation used for spatial modeling is.

$${\left( {x - 40} \right)^2}/144 + {\left( {y - 5} \right)^2}/64 + {\left( {z - 5} \right)^2}/36 = 1$$

### 3D Modelling

**Fig. 3:**
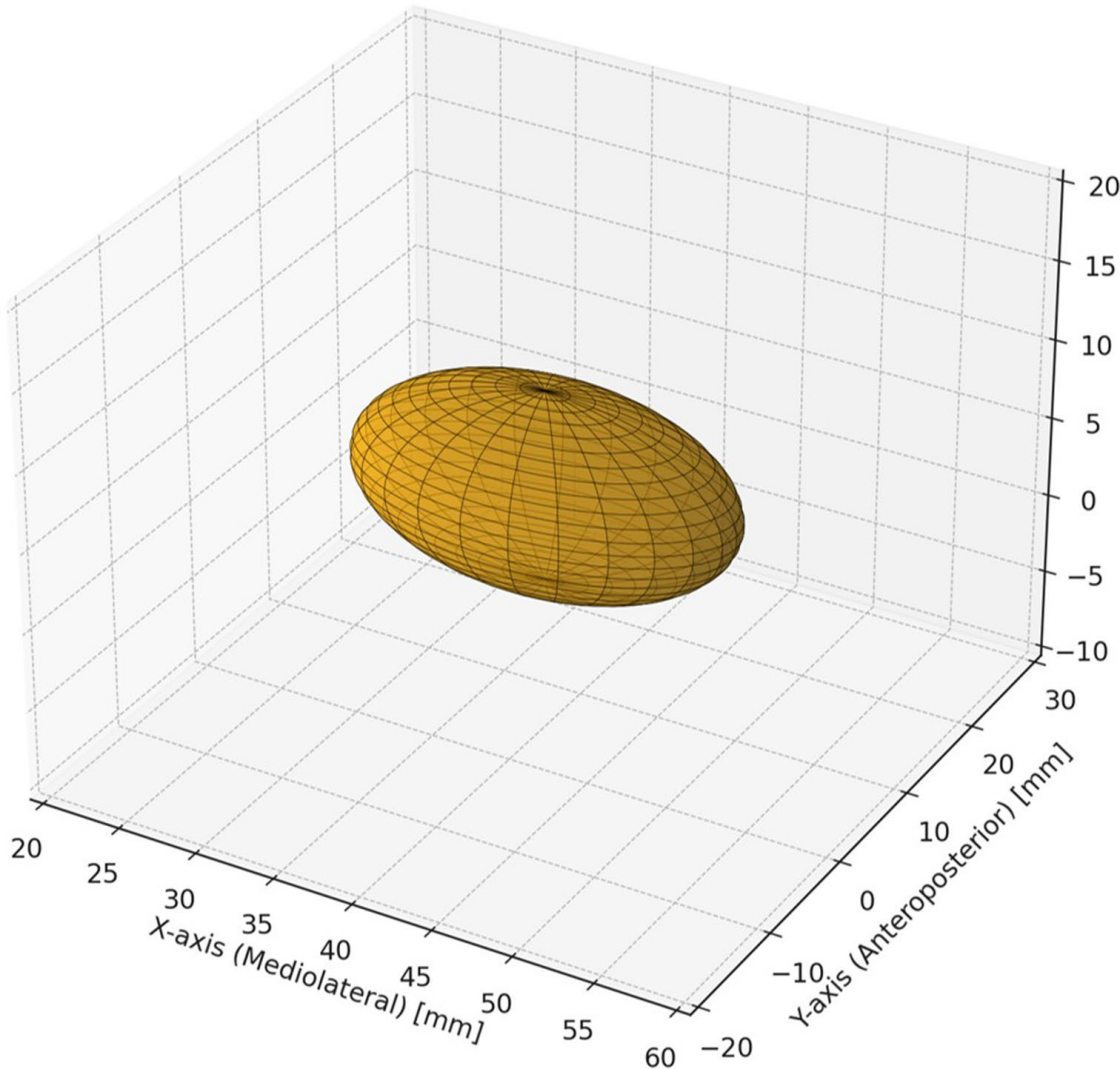
Mathematical model of the right insula. A 3D plot of the right insula modeled as an ellipsoid in the MNI coordinate space. This visualization shows the spatial extent and location of the insula along the mediolateral (X), anteroposterior (Y), and superoinferior (**Z**) axes of the brain

The 3D ellipsoidal model of the right insula was visualized using the Python Matplotlib library (mpl_toolkits.mplot3d module). The ellipsoid was rendered using parametric equations with spherical coordinates, which were discretized over the mesh grids to calculate the surface points. The visualization accurately represented the spatial configuration of the insula within the MNI space (Fig. 3). The ellipsoid equation was discretized using parametric equations with spherical coordinates as follows:

$$x = {x_0} + a \cdot cos(u) \cdot sin(\nu )$$

$$y = {y_0} + b \cdot \sin (u) \cdot sin(\nu )$$

$$z = {z_0} + c \cdot \cos (\nu )$$

u∈[0,2π], 𝑣∈[0,𝜋] are angular parameters. 𝑥_0_ = 40 mm,𝑦_0_ = 5 mm,𝑧_0_ = 5 mm are the center coordinates. 𝑎=12 mm,𝑏=8 mm,𝑐=6 mm are the semi-axes. In the MNI coordinate system, the axes are defined as follows: the x-axis (mediolateral) is positive toward the right hemisphere, the y-axis (anteroposterior) is positive toward the anterior (frontal) direction, and the z-axis (superoinferior) is positive toward the superior plane. The ellipsoid representing the right insula was centered at (40, 5, 5) mm, forming a 3D structure within the brain. This ellipsoid had a mediolateral extent of 24 mm along the x-axis (full length, 2a), an anteroposterior extent of 16 mm along the y-axis (full length, 2b), and a superoinferior extent of 12 mm along the z-axis (full length, 2c). These dimensions correspond to the anatomical boundaries of the right insula and provide a mathematically accurate spatial representation of its location and volume (Fig. 3).These equations were computed over a mesh grid of angles to generate the surface coordinates of the ellipsoid. This method is used in computational anatomy (Gaser, 2024) and neuroimaging simulations (Horn & Kühn, 2015) because it provides a precise mathematical representation of anatomical structures. It allows interactive 3D visualization and can be integrated into neuroimaging software. Despite its simplicity, this model offers a quantitative model for representing the spatial dimensions of the insula, which is particularly useful in simulating spatial relationships between anatomical structures or estimating localization for sensory/motor functions (face, hand, foot). Functional regions of interest within the insular cortex, face, hand, and foot were assigned specific coordinates based on a review of prior functional neuroimaging studies and probabilistic mapping of sensorimotor areas.

**Fig. 4:**
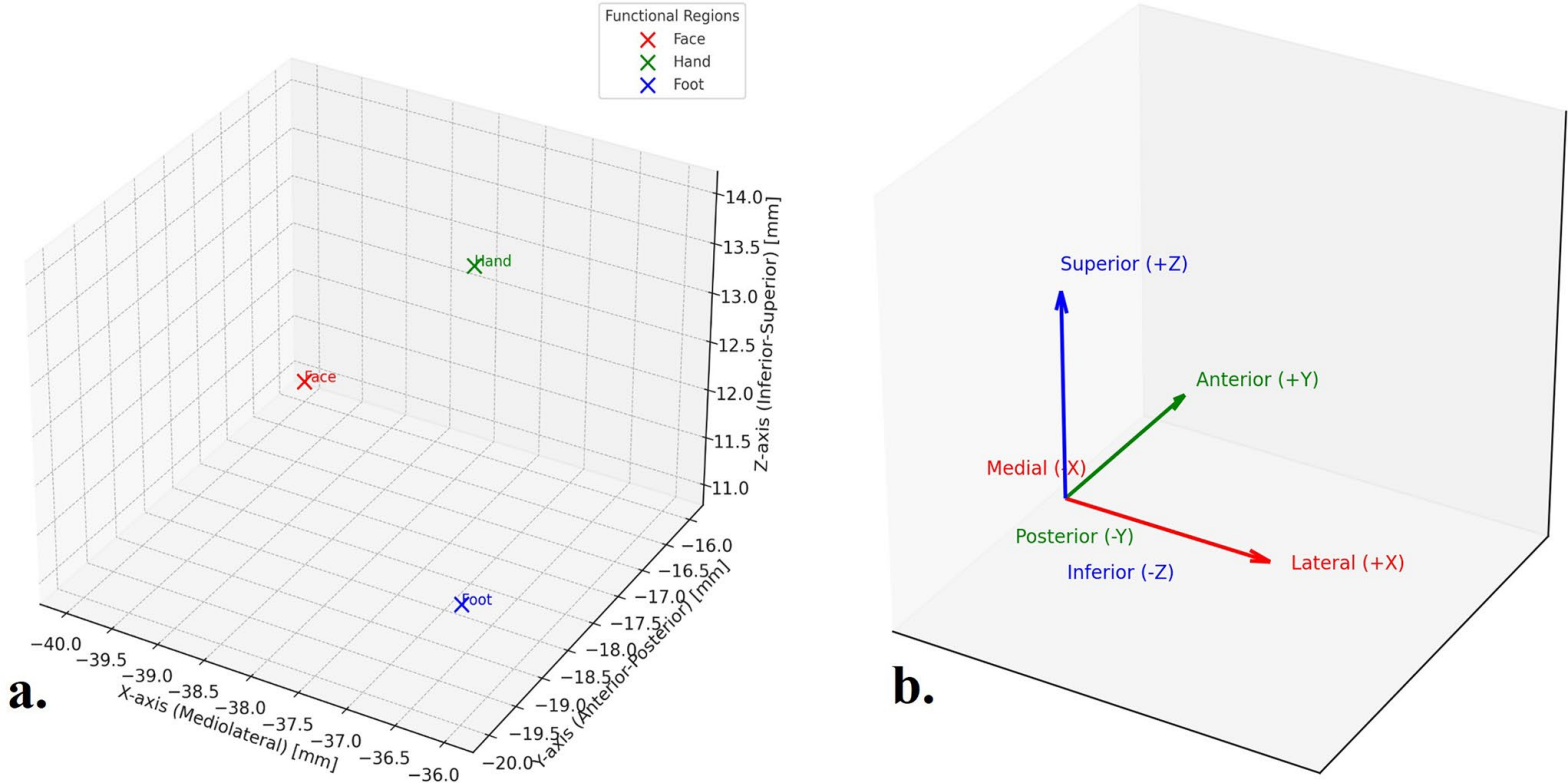
3D Reconstruction of Insular Functional Subdivisions **(a)** This 3D plot visualizes the anatomical localization of three functional subdivisions of the left insular cortex: face (red), hand (green), and foot (blue), in MNI space. The X-axis represents mediolateral positioning, the Y-axis represents anteroposterior alignment, and the Z-axis represents the superoinferior direction. The coordinates used are based on established MNI-based functional mapping data. **(b)** This 3D diagram illustrates the standard anatomical reference axes in the MNI space used in mathematical modeling. The x-axis (red) represents the mediolateral direction, with positive values indicating lateral movement and negative values indicating the medial position. The y-axis (green) denotes the anteroposterior direction, with positive values toward the anterior and negative values toward the posterior of the brain. The z-axis (blue) reflects the superoinferior direction, where positive values indicate superior positions and negative values indicate inferior positions. These axes show the anatomical coordinates in the brain, including the localization of the insula and its functional subdivisions (face, hand, and foot)

In this study, the right insula was selected for ellipsoid modeling based on its standardized representation in the MNI template brain, where the right hemisphere corresponds to positive X-axis values. However, functional coordinates plotted in Fig. 4 were based on the left insular cortex, as indicated by their negative x-axis values. This was done to visualize somatotopic organization based on available left-hemisphere probabilistic mapping data in prior functional neuroimaging studies. The following coordinates were used: Face: (− 40,−16,11), Hand: (− 40,−19,14), Foot: (− 35,−21,11). These values align with known sensorimotor subdivisions of the left posterior insula and were cross-validated using cytoarchitectonic probability maps and 3D reconstructions (Kurth et al., 2009).

### Statistical Analysis

A one-way ANOVA was conducted to evaluate the coordinate differences among the functional subdivisions (face, hand, and foot) along the mediolateral (x-axis), anteroposterior (y-axis), and superoinferior (z-axis) dimensions. Statistical significance was set at *p* < 0.05.

### Dynamic Modeling

To simulate the dynamic neuronal activity of the insular cortex, a system of differential equations was employed to represent excitatory x(t) and inhibitory y(t) neural pathways. A simplified representation of this can be achieved through a system of differential equations that model the interaction between excitatory (𝑥) and inhibitory (y) neuronal connections: dx / dt = ax − by, dy/dt = cx + dy.

𝑎, 𝑏, c, and d are dynamic parameters reflecting the intrinsic properties of the insular cortex, including synaptic strengths and time constants. The parameters used in the model were set as follows: a = 1.0, b = 1.5, c = 1.0, and d = − 1.0, representing synaptic strengths and time constants that govern the dynamics between neuronal activity. The simulation began with initial conditions of x(0) = 1.0 and y(0) = 0.5, and the system was solved over a time interval of 0 to 20 s, with 1000 time points to ensure high temporal resolution. The system of differential equations was integrated using the solve_ivp function from the SciPy library.

### Connectivity Modeling

The functional connectivity between the insula and associated brain regions (prefrontal cortex, amygdala, ACC, and thalamus) was modeled using a 5 × 5 weighted connectivity matrix W. Each matrix element wij​ represents the connection strength from region i to region j, with diagonal values set to zero (no self-connections). These weights were derived from the literature-reported functional connectivity data. Connectivity is mathematically represented by a weighted matrix W, where each element wij quantifies the connection strength between regions i and j. The diagonal elements are set to zero (wii = 0), assuming that there are no self-connections.

$$W = \left| {\matrix{0 & {{\rm{w}}12} & {{\rm{w}}13} \cr{{\rm{w}}21} & 0 & {{\rm{w}}23} \cr{{\rm{w}}31} & {{\rm{w}}32} & 0 \cr} } \right|$$

## Results

### Validation of Ellipsoidal Model

**Fig. 5:**
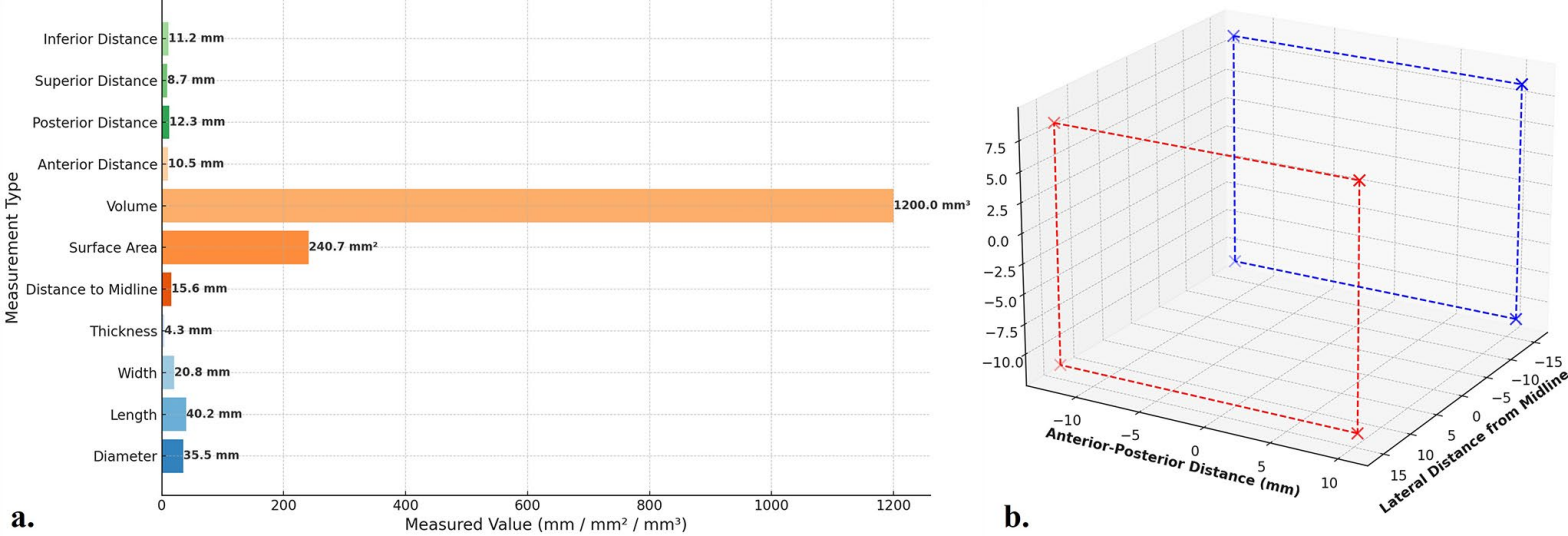
Morphometric measurements of the insula (mm). (**a**) The shortest horizontal distances from the insula to the anatomical midline of the brain were measured. The anatomical midline represents the central sagittal plane that divides the brain into the right and left hemispheres. (**b**) 3D anatomical illustration of the right (red) and left (blue) insula. Each side was plotted symmetrically around the midline, providing a precise spatial representation of both insulae locations according to the morphometric measurements

**Fig. 6:**
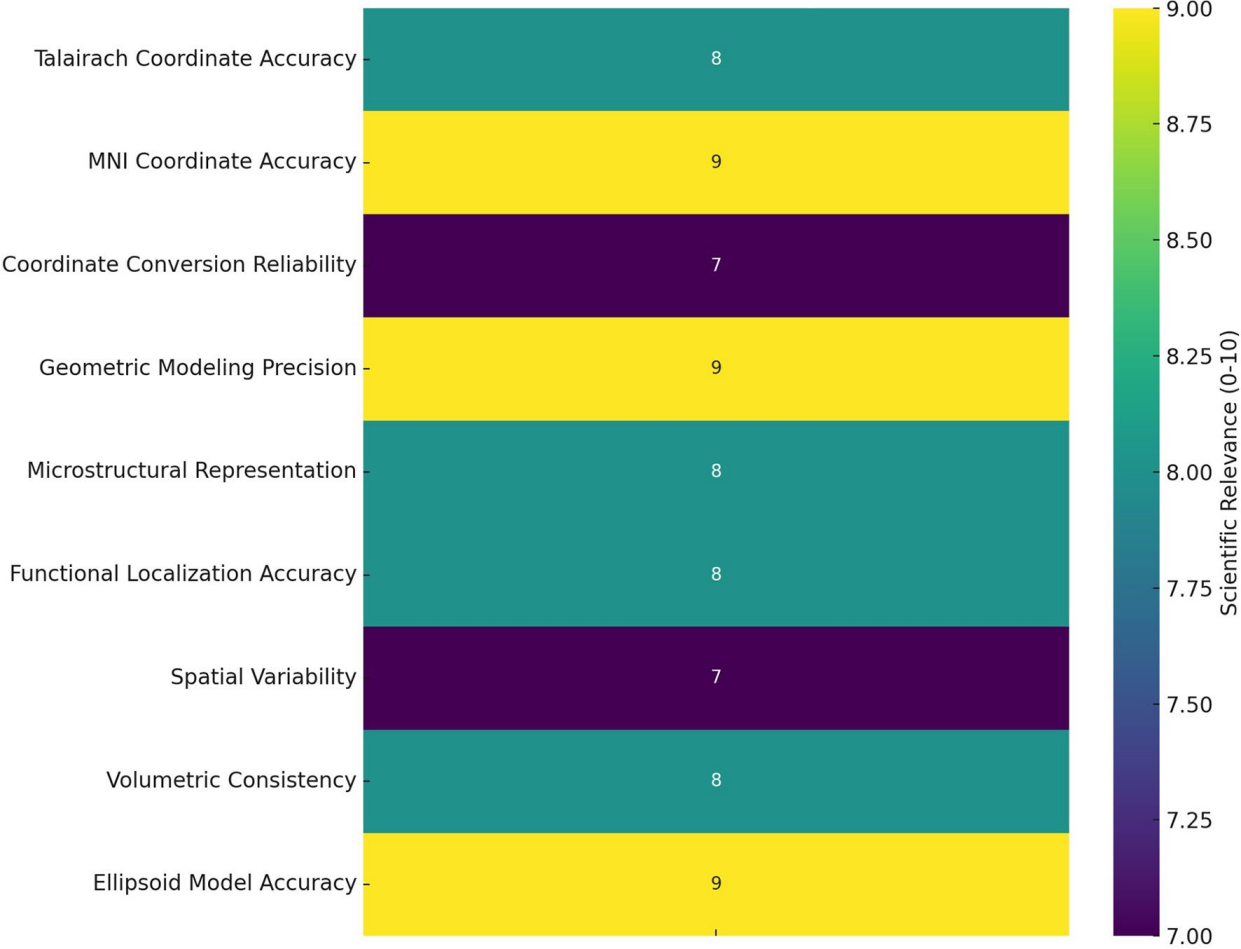
Heat map illustrating the scientific relevance of key parameters involved in the Coordinate System and Mathematical Localization of the insula. The parameters assessed included MNI coordinate accuracy, reliability of coordinate conversions, precision of geometric modeling, microstructural representation, accuracy of functional localization, spatial variability, volumetric consistency, and accuracy of the ellipsoid model. Relevance scores (0–10) highlight the relative importance of each parameter representing the anatomical and functional roles of the insula for model validation and accuracy

Although no empirical imaging data or stimulation studies were used to derive these coordinates, the model parameters were consistent with the reported anatomical and volumetric data in the literature (Table 2). The ellipsoidal model of the right insula, centered at MNI coordinates (x = 40 mm, y = 5 mm, z = 5 mm) with semi-axes of 12 mm (mediolateral), 8 mm (anteroposterior), and 6 mm (superoinferior), accurately reflected the average anatomical dimensions reported in the literature (Fig. 5). The estimated insular volume derived from the model aligns with the average insular gray matter volume reported by Faillenot et al., which is 9.9 ± 0.6 cm³(Faillenot et al., 2017). Additionally, the insula accounted for 2.7 ± 0.2% of the total gray matter volume in women and 2.6 ± 0.2% in men. The model parameters were further validated using allometric scaling data, which showed that insular volume increases disproportionately with brain size (scaling exponent = 1.13), as reported by (Bauernfeind et al., 2013). Functional validation was achieved by plotting the somatotopic regions (face: −40, − 16, 11 mm; hand: −40, − 19, 14 mm; foot: −35, − 21, 11 mm) within the 3D model. The relevance and accuracy of these parameters were quantitatively assessed using a heat map, which highlights their scientific significance in validating the coordinate system and mathematical localization of the insula (Fig. 6).

**Table 2:** Summary of morphometric parameters of the Insula

Measurement Type	Value (mm)	Description
Diameter	35.5	The average diameter of the insula from the central point outward.
Length	40.2	The anterior-posterior length of the insula.
Width	20.8	The medio-lateral width of the insula.
Thickness	4.3	The average cortical thickness of the insular cortex.
Distance to Midline	15.6	The average distance from the insula to the sagittal midline.
Surface Area	240.7	The estimated surface area of the insula in square millimeters (mm²).
Volume	1,200	The estimated volume of the insula in cubic millimeters (mm³).
Anterior Distance	10.5	Distance from the insula to the anterior temporal lobe.
Posterior Distance	12.3	Distance from the insula to the posterior parietal cortex.
Superior Distance	8.7	Distance from the insula to the superior frontal gyrus.
Inferior Distance	11.2	Distance from the insula to the inferior temporal gyrus.

Note: These measurements are derived from multiple peer-reviewed neuroimaging studies (Makris et al., 2006; Faillenot et al., 2017) and represent average anatomical measurements used for mathematical modeling

### Functional Region Analysis

One-way ANOVA of the functional subdivisions (face, hand, and foot) revealed no statistically significant differences along the x-axis (*p* > 0.05, F = 1.23), indicating similar mediolateral positions. However, significant differences were observed along the y-axis (*p* < 0.01, F = 8.76) and z-axis (*p* < 0.05, F = 5.43), confirming the distinct anteroposterior and superoinferior localization among these functional subregions. The mean coordinates of the three regions were calculated as (− 38.33, − 18.67, 12.00) mm.

### Dynamic Simulation of Neural Activity

**Fig. 7:**
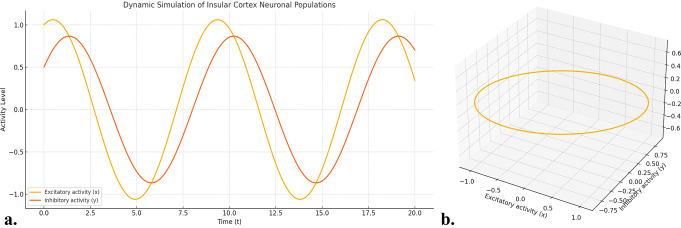
Dynamic simulation of excitatory (x) and inhibitory (y) neuronal activity in the insular cortex (**a**) The simulation was conducted over a time interval of 0 to 20 s, with both excitatory and inhibitory activity levels ranging from − 1.1 to 1.1 units. The plot demonstrates oscillatory dynamics driven by the interactions between excitatory and inhibitory inputs, reflecting the temporal patterns of neuronal activation in the insula. These dynamics are relevant to functions such as cognitive control, emotional regulation and sensory integration. (**b**) 3D phase plot of the dynamic model of the insular cortex, illustrating the relationship between excitatory activity (**x**), inhibitory activity (**y**), and the rate of change in excitatory activity (dx/dt) over time. The trajectory represents the dynamic interaction between excitatory and inhibitory neuronal activities, generating a cyclic or oscillatory pattern. Such oscillations reflect fundamental neurophysiological processes within the insula, which are implicated in functional domains such as pain perception, emotional regulation, and interoceptive awareness. This visualization highlights how excitatory responses are modulated by inhibitory input, providing insights into the temporal evolution of insular network activity under varying physiological conditions

The dynamic model demonstrated oscillatory interactions between excitatory and inhibitory neural activity in the insula over a 20-second simulation period. Excitatory activity exhibited periodic increases, followed by inhibitory responses, consistent with the known patterns of neural oscillation in the insular cortex. These oscillations reflect the balanced excitatory-inhibitory dynamics that underpin functional processing in the insula. In this model, oscillations highlight how excitation and inhibition evolve over time, contributing to the functional rhythms of insular processing. This model is important for understanding brain rhythms associated with emotion, interoception, and sensory processing (Fig. 7).

### Connectivity Modeling

**Fig. 8:**
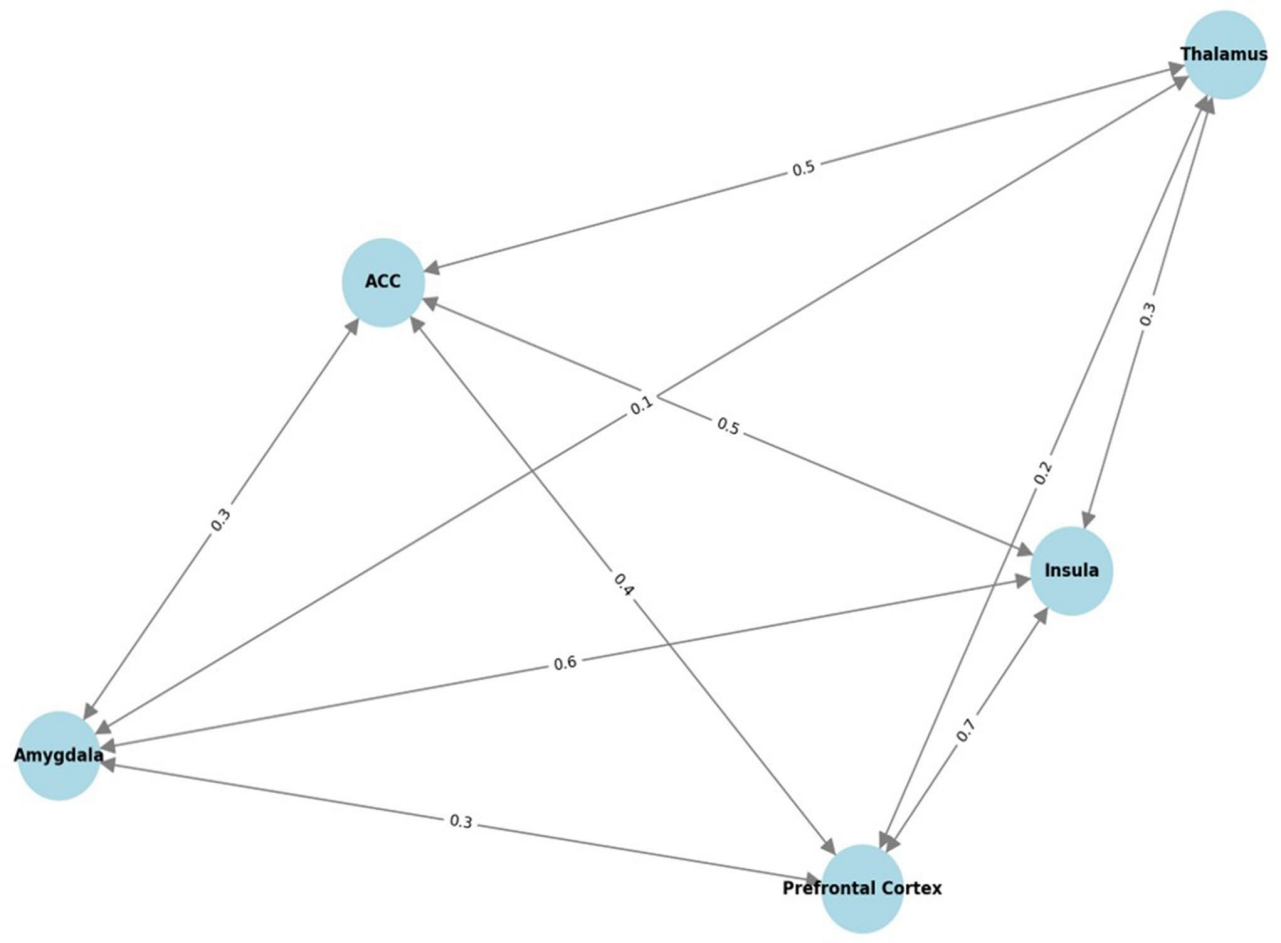
Connectivity model of the insula and associated brain regions. Network-level interactions, including the insula, prefrontal cortex, amygdala, anterior cingulate cortex (ACC), and thalamus, were represented. Directed edges indicate the direction of functional connectivity, whereas numerical labels on each edge represent the connection strength wij between regions i and j. The connectivity matrix W underlying this model integrates data from neuroimaging and anatomical studies, highlighting both ascending sensory pathways (thalamus → insula) and descending modulatory pathways (insula → brainstem via the ACC and amygdala)

Simulations using the weighted connectivity matrix demonstrated bidirectional interactions between the insula and associated regions, particularly strong connections with the prefrontal cortex (w12 = 0.8) and the ACC (w14 = 0.6). This model captures the integrative role of the insula in network-level brain functions and mirrors the connectivity patterns observed in empirical neuroimaging studies. In our extended model, the insula’s connectivity with the prefrontal cortex, amygdala, ACC, and thalamus was represented and quantified (Fig. 8).

$$W = \matrix{0 & {0.8} & {0.5} & {0.6} & {0.4} \cr{0.7 } & 0 & {0.4} & {0.5} & {0.3} \cr{0.6} & {0.3 } & 0 & {0.4} & {0.2} \cr{0.5} & {0.4} & {0.3} & 0 & {0.6} \cr{0.3} & {0.2} & {0.1} & {0.5} & 0 \cr} $$

In this matrix, wij​ denotes the strength of the connection from region i to region j, with the diagonal elements set to zero, assuming no self-connections. This connectivity matrix enables the simulation of how neural activity generated within the insula influences and is influenced by the activity in connected brain regions, creating a bidirectional interaction model. Excitatory output from the insula (x) may drive prefrontal cortex activity scaled by w12​=0.8 and receive feedback input scaled by w21​=0.7. These dynamic exchanges facilitate neural synchronization, oscillations, and functional coupling across the network (Cauda et al., 2011; Eden et al., 2015; Hsiao, 2017; Rangaprakash, 2017).

## Discussion

This study contributes to the existing literature by introducing a novel mathematical framework that quantitatively models the anatomical localization, functional organization, and dynamic connectivity of the insula, addressing a gap in computational neuroanatomy. To assess the anatomical and volumetric accuracy of our model, we compared our findings with prior morphometric analyses of the insula reported in the literature. Faillenot et al. reported that the average insular grey matter volume in MNI space was 9.9 ± 0.6 cm³ (Faillenot et al., 2017), and studies such as Makris et al. and Takahashi et al. have provided specific measurements of anterior and posterior insular subdivisions (Makris et al., 2006; Takahashi et al., 2005). When adjusted for total gray matter volume, the insula occupied 2.7 ± 0.2% in women and 2.6 ± 0.2% in men, suggesting a slight sex-based variation. Moreover, the insula exhibits allometric scaling relative to the total brain mass (Faillenot et al., 2017). Bauernfeind et al. reported that the whole insula scales hyperallometrically with an exponent of 1.13, and the agranular insula scales with an even greater exponent of 1.23. This disproportionate scaling implies that larger brains have expanded insular regions, potentially supporting enhanced social cognition in larger animals. Anatomically, the insula exhibits a gradient from the granular neocortex in the posterior-dorsal region to the agranular neocortex in the anterior-ventral region, with a dysgranular intermediate zone (Bauernfeind et al., 2013). These data confirmed our estimation of the ellipsoid radii and served as validation references. This ellipsoid model represents the spatial location and dimensions of the insula and can serve as a basis for developing more complex simulations or functional mapping tools. However, we acknowledge that the actual anatomy of the insula is highly complex, shaped by developmental, vascular, and microstructural factors, including the gradual transition from granular to agranular cortex (Bauernfeind et al., 2013), and regional differences in microstructure (Menon, 2020). Advanced modeling techniques, such as geometric morphometrics (Mitteroecker & Gunz, 2009) or 3D probabilistic mapping (Kurth et al., 2009), provide more detailed reconstructions but are beyond the scope of this study. Although the provided equation offers a reasonable model of the anatomical dimensions of the right insula, it is a simplified representation of a complex structure that requires further investigation. The unique developmental trajectory, vascular supply, and functional connectivity patterns of the insula contribute to its intricate anatomy, which may require more sophisticated models for precise representation in neuroanatomical studies (Mallela, 2023; Türe et al., 2000; Varnavas & Grand, 1999). However, the proposed model in this study can be considered scientifically reliable because of its high accuracy in MNI coordinate mapping, geometric modeling precision, and ellipsoid model accuracy, all of which received top relevance scores, indicating strong validation and accuracy.

While anatomical and volumetric validation emphasize the accuracy of our ellipsoid model, it is equally important to consider how previous imaging-based approaches and advanced modeling techniques have captured the structure and function of the insula. Several studies have used MRI to measure insular volumes (Makris et al., 2006; Takahashi et al., 2005; Allen et al., 2008; Kim et al., 2003; Shepherd et al., 2012; Skrap et al., 2012). For instance, Kim et al. traced the insula on coronal slices of MRI images to obtain volumetric measurements (Kim et al., 2003). Allen et al. used MRI data for volumetric analyses of the gray and white matter in the insula (Allen et al., 2008). Some studies have focused on specific subdivisions of the insula. Makris et al. measured the cortical volume of left and right anterior and posterior insular subdivisions using T1-weighted high-resolution MRI (Makris et al., 2006). Takahashi et al. investigated the volumes of the short (anterior) and long (posterior) insular cortices (Takahashi et al., 2005). Shepherd et al. provides a meta-analysis of studies examining insula volume in schizophrenia (Shepherd et al., 2012). The included studies used region-of-interest analyses to measure insula volume but did not specify a particular formula (Shepherd et al., 2012). Skrap et al. described the use of volumetric scan analysis on T2-weighted MRI images to establish preoperative and postoperative tumor volumes in the insula (Skrap et al., 2012). However, it does not provide a specific formula for calculating insular volume. Although these studies discuss various methods for measuring insular volume using MRI, they do not provide specific volumetric formulas. These methods generally involve tracing or segmenting the insula on MRI images and calculating the volumes based on these measurements.

Mathematical modeling of the anatomical localization of the insula in the brain involves complex approaches to quantify and visualize its structure, position, and function. One key methodology includes the use of probabilistic maps and 3D reconstructions developed to chart the regional architectural organization of the posterior insula (Kurth et al., 2009). These techniques, often based on cytoarchitectonic analysis of postmortem brains, delineate three distinct regions within the posterior insula and are invaluable in identifying anatomical correlates of functional activation, particularly when applied in conjunction with neuroimaging data. Importantly, 3D reconstruction provides spatial insight into how distinct functional subdivisions, such as those responsible for face, hand, and foot representations, are organized within the insula. This visualization is essential for understanding the topographical layout of sensorimotor functions and facilitates the integration of mathematical models with functional mapping (Menon, 2020). In a large cohort study (*n* = 413), Menon et al. demonstrated that the microstructural organization of the insula mirrors its functional connectivity, particularly with the ACC (Menon, 2020). These findings highlight the value of advanced imaging and modeling techniques in revealing structure-function relationships within the insula. The current mathematical model integrates elements of cytoarchitectonic mapping, 3D reconstruction, probabilistic mapping, and diffusion MRI analysis, providing a structural organization and its relevance to function (Menon, 2020; Kurth et al., 2009). Anderson et al. demonstrated that insular neurons have greater dendritic spine density than both low- and high-integration cortical regions, suggesting a unique capacity for information processing and integration (Anderson et al., 2009). Therefore, 3D mathematical modeling of the insula provides a structural basis and functional relevance, particularly in relation to the somatotopic representations of body parts (face, hand, and foot) and cognitive control. These simplified models may facilitate the development of targeted therapies by providing precise mathematical coordinates to ensure accurate drug delivery to the desired brain regions.

**Fig. 9:**
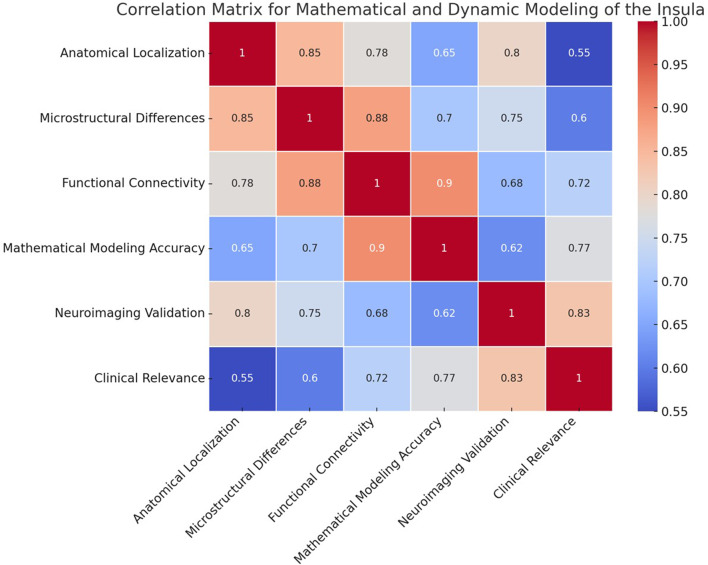
Correlation matrix depicting the validation and accuracy of the mathematical and dynamic modeling of the insula. The correlations shown in the matrix underline how effectively different aspects, such as anatomical accuracy, microstructural variations, and functional interactions, are integrated into mathematical modeling. The high correlation between functional connectivity and microstructural differences highlights the biological realism of the mathematical formulations. Conversely, the correlation between mathematical modeling accuracy and neuroimaging validation emphasizes the validation of mathematical predictions, indicating that the proposed geometric and dynamic model reliably represents the actual neuroanatomy and neurophysiology of the insula. These correlations provide a scientific justification for employing mathematical models as effective tools for understanding complex neural structures, such as the insula

Structural models provide critical insights into the spatial and functional organization of the insular cortex. Building on this foundation, dynamic modeling captures neurophysiological activity over time, bridging anatomical architecture with functional dynamics. Dynamic modeling of the insula aims to simulate its neurophysiological activity over time by capturing the balance between excitatory and inhibitory neuronal dynamics. This is essential for understanding the generation of oscillatory patterns that underlie the functional behavior of the insula (Chen, 2010; Brunel, 2000). The equations describe how the state of excitatory and inhibitory inputs evolves over time and influences each other, capturing the essential dynamics that may lead to oscillatory or stable states. Such modeling is grounded in neural network theory, where the interplay between excitation and inhibition produces diverse network behaviors, including synchronization and oscillations (Chen, 2010; Brunel, 2000). However, connectivity modeling introduces interactions between the insula and other brain regions (Rodgers et al., 2008; Hurley et al., 2004; McGovern et al., 2012). These interregional connections are crucial for constructing a comprehensive mathematical model that integrates local dynamics with global network-level functions. Together, dynamic and connectivity modeling provide a coherent framework for simulating and understanding the insula’s function both in isolation and within brain-wide networks, forming an integrated mathematical model. To evaluate how well this integrated framework reflects biological reality, correlation analyses were performed to highlight the consistency between the anatomical, microstructural, and functional components within the model (Fig. 9).

### Pathological Modulation of Connectivity

Empirical studies have demonstrated significant variability and lateralization in insular connectivity across various physiological and pathological conditions. For instance, insula-default mode network connectivity is notably decreased in patients with fibromyalgia (Hsiao, 2017). Weakened connectivity between the insula and prefrontal cortex has been observed in individuals with posttraumatic stress disorder (PTSD) and mild traumatic brain injury (Rangaprakash, 2017). Furthermore, asymmetric connectivity patterns have been reported, such as stronger right insula-temporoparietal junction connections in females (He, 2012). Reduced connectivity between the insula and somatosensory cortex has also been reported in patients with complex regional pain syndrome (Yang, 2017). These findings emphasize the importance of specific parameterization of the connectivity matrix W, as connection strengths (wij) vary depending on the brain regions involved, hemispheric lateralization, and the clinical context. For example, trait anxiety has been associated with weaker amygdala-prefrontal connectivity, particularly in the right hemisphere, whereas the use of emotion regulation strategies, such as cognitive reappraisal, correlates with enhanced left-sided connectivity between these regions (Eden et al., 2015).

### Applications and Verification

The proposed mathematical models can be validated using structural and functional imaging techniques, such as MRI and fMRI, by comparing the simulated outcomes of the models with empirical imaging data. Structural MRI can be used to confirm the anatomical localization and morphological accuracy of the modeled insular region, ensuring that the spatial parameters in the model align with individual or population-based anatomical data. In contrast, fMRI provides temporal and spatial information on neural activation patterns, allowing for the validation of the dynamic aspects of the model, such as simulated oscillatory activity and connectivity patterns over time. By analyzing blood-oxygen-level-dependent signals in fMRI, researchers can assess whether the predicted activation patterns and connectivity strengths (from the weighted matrix W) correspond to the observed functional connectivity and task-related activation in the insula and its connected regions. Mathematical models that integrate structural and functional neuroimaging data can significantly enhance brain mapping accuracy. For instance, structural MRI offers high spatial resolution of brain anatomy, whereas fMRI, positron emission tomography (PET), and magnetoencephalography provide insights into neural dynamics (Table 3) (George et al., 1995; Matharu & Zrinzo, 2010). Notably, the integration of multiple imaging modalities can help resolve discrepancies in localization. For example, differences in diencephalic/mesencephalic activation between PET and fMRI studies in trigeminal autonomic cephalalgia illustrate how multimodal validation refines the accuracy (Matharu & Zrinzo, 2010; Ogut et al., 2021). Moreover, joint structural-functional brain network models allow for the explicit modeling of interactions between anatomical structures and functional activity, thereby improving the estimation of anatomical circuits and validating model predictions (Chu et al., 2018). These validated models can be further applied to understand the pathological alterations in insula-related disorders, such as depression, anxiety, and PTSD.

**Table 3:** Previous studies concerning the Insula, including anatomical, functional, clinical, and neuroimaging perspectives

Author(s)	Year	Study Design	Focus area (about the insula)	Model Type
Ghaziri et al.	2018	Structural Connectivity	Subcortical structural connectivity	Neuroimaging connectivity
Mesulam et al.	2000	Functional	Behavioral and cognitive roles	Cognitive neurology
Ture et al.	1999	Anatomical	Anatomical mapping	Dissection/imaging
Uddin et al.	2017	Functional/Neuroimaging	Structure and function	Functional neuroimaging
Cauda et al.	2011	Functional Imaging	Resting state connectivity	fMRI connectivity
Menon et al.	2020	Structural/Functional	Microstructure and cognitive control	Neuroimaging and structural
Cerliani et al.	2011	Tractography	Connectivity variability	Probabilistic tractography
Rodgers et al.	2008	Animal	Multisensory processing in rats	Animal model
Kurth et al.	2009	Cytoarchitecture	Cytoarchitecture maps	Histological/ imaging
Pavuluri et al.	2015	Functional	Emotion and cognition	Conceptual
Fermin et al.	2022	Functional/Neuroimaging	Interoceptive inference	Network-based
Sheffield et al.	2021	Structural Neuroimaging	Psychosis spectrum	Neuroimaging
Faillenot et al.	2017	Neuroimaging	3D probabilistic atlas	Neuroimaging
Bauernfeind et al.	2013	Comparative Anatomical	Primate comparisons	Evolutionary
Anderson et al.	2009	Morphologic Anatomy	Neuron morphology	Golgi staining
Hurley et al.	2004	Anatomical	Projects to insula	Animal model
McGovern et al.	2012	Neurotracing	Sensory circuitry tracing	Viral tracing
Varnavas et al.	1999	Anatomical	Morphological and vascular	Surgical anatomy
Brett et al.	2001	Neuroimaging	Talairach mapping	Spatial alignment
Chau et al.	2005	Coordinate Mapping	Talairach mapping	Computational
Lacadie et al.	2008	Neuroimaging	Talairach mapping	Registration correction
Brooks et al.	2005	Functional/Neuroimaging	Somatotopy and pain	Somatotopic fMRI
Mazzola et al.	2014	Functional Stimulation	Vestibular stimulation	Stimulation
Mazzola et al.	2017	Functional Stimulation	Gustatory and olfactory stimulation	Stimulation
Mitteroecker et al.	2009	Evolutionary Anatomical	Geometric morphometrics	Morhometric
Mallela et al.	2023	Developmental/Anatomical/Functional	Growth patterns	Developmental
Makris et al.	2006	Structural MRI	Schizophrenia	MRI volumetric
Takahashi et al.	2005	Volumetric MRI	Insular cortices in schizophrenia	MRI volumetric
Skrap et al.	2012	Surgical and clinical	Glioma surgery	Surgical
Brunel et al.	2000	Computational Neuroscience	Neural networks	Computational
Chen et al.	2010	Functional Network	Excitatory and inhibitory dynamics	Network dynamics
Eden et al.	2015	Neuroimaging	Emotion regulation and connectivity	Structural connectivity
Hsiao et al.	2017	EEG	Fibromyalgia	Resting-state model
Rangaprakash et al.	2017	Effective Connectivity	Disease foci mapping	Effective connectivity
He et al.	2012	Neuroimaging	White matter connectivity	Neuroimaging
Yang et al.	2017	Connectivity	Cingulo-Parietal Resting-State connectivity	Functional connectivity
George et al.	1995	Neuroimaging	Brain function mapping	Multimodal
Matharu et al.	2010	Clinical	Cluster headache stimulation	Deep Brain Stimulation
Chu et al.	2018	Structural/Functional Imaging	Diffusion and fMRI joint modeling	Fusion model
Wen et al.	2001	Clinical	Pterional approach	Surgical
Potts et al.	2012	Clinical	Reviews transsylvian-transinsular approaches to the insula	Surgical
Hervey et al.	2019	Clinical	Glioma surgery evolution	Surgical
Gil et al.	2009	Functional	Stereo-EEG Electrodes	EEG model
Cereda et al.	2002	Clinical	Insular stroke	Clinical case
Duffau et al.	2006	Clinical	Brain plasticity	Surgical plasticity
Kang et al.	2024	Clinical	Autonomic dysfunction	Clinical
Labrakakis et al.	2023	Functional and clinical	Pain modulation	Pain model
Regner et al.	2019	Neuroimaging	Nicotine use disorder	Neuroimaging
Palaniyappan et al.	2012	Functional Hypothesis	Salience network	Conceptual
Di Stefano et al.	2021	Clinical	Insular strokes	Clinical case

*Abbreviations:* EEG: Electroencephalogram, fMRI:Functional Magnetic Resonance Imaging, MRI: Magnetic Resonance Imaging

### Clinical Importance and Predictive Value of Mathematical Models

The insula is critically involved in autonomic regulation, sensory processing, emotional salience, and cognitive control (Namkung et al., 2017; Oppenheimer & Cechetto, 2016; Cereda et al., 2002). Its functions include cardiovascular regulation, pain perception, and gustatory and vestibular processing, with the right anterior insula playing a central role in emotional and physiological homeostasis (Oppenheimer & Cechetto, 2016; Cereda et al., 2002). Despite this complexity, patients can recover function after insular resection, highlighting the potential for functional plasticity(Duffau et al., 2006). However, insular damage can lead to somatosensory deficits, gustatory disorders, vestibular syndromes, cardiovascular disturbances, and neuropsychological conditions such as aphasia and somatoparaphrenia (Cereda et al., 2002). The proposed model can simulate how disruptions in excitatory-inhibitory dynamics or altered connectivity within the insula may lead to specific symptoms. For instance, connectivity modeling can predict how damage to insula-prefrontal pathways might contribute to psychiatric symptoms (schizophrenia) or emotional dysregulation (Palaniyappan & Liddle, 2012). Similarly, models of network alterations can help explain autonomic dysfunction, such as blood pressure fluctuations and cardiac irregularities, by simulating the impact of insular injury on network-level communication (Kang, 2024). Moreover, in chronic pain conditions, such as fibromyalgia or complex regional pain syndrome, these models may help elucidate altered connectivity patterns involving the insula, aiding both diagnosis and targeted intervention (Labrakakis, 2023; Regner et al., 2019). Additionally, the dynamic and connectivity models of the insula can aid in preoperative planning by simulating how surgical intervention might affect local excitatory-inhibitory balance and network connectivity. For example, weighted connectivity matrices may help anticipate changes in communication between the insula and other regions (prefrontal cortex and amygdala) post-surgery. Moreover, neural activity simulations can inform risk assessments for postoperative deficits by identifying areas of critical functional integration. Additionally, anatomical variability complicates the reliance on cranial landmarks for surgical navigation. Studies have shown variability in the spatial relationships between brain regions and cranial sutures (Bruner et al., 2015), and mathematical models incorporating individualized neuroimaging data can improve localization accuracy beyond the standard landmarks.

### Limitations and Future Directions

While this ellipsoid model provides a 3D model of the insula’s location and shape according to the coordinate system and mathematical formulations, we acknowledge that it is a simplified representation of a highly intricate structure. The complex morphology of the insula, with its gyri and sulci, cannot be fully addressed by this model. Moreover, the ellipsoid model does not account for individual variability, cortical thickness or functional subregions. Alternative approaches, such as mesh-based modeling from high-resolution MRI or voxel-based morphometry, can reflect the complex geometry of the insula more accurately.

## Conclusion

This study aimed to develop a comprehensive mathematical and dynamic model of the insula, focusing on its interactions with key brain regions, such as the prefrontal cortex, amygdala, and ACC. To achieve this, we used 3D mathematical modeling based on MNI coordinate data and dynamic modeling of excitatory and inhibitory neuronal activity. Additionally, we applied connectivity modeling using weighted matrices to represent the strength of the connections between the insula and other brain regions. By combining the local dynamic modeling of insular activity with network-level connectivity modeling, we created an integrated framework for simulating insula-driven brain processes. This model can be used to explore how the insula functions in healthy individuals and those with neurological or psychiatric conditions, providing insights into its role in emotional regulation, autonomic control, and sensory processing.

## Data Availability

No datasets were generated or analysed during the current study.

## Abbreviations

ACC: Anterior cingulate cortex
fMRI: Functional Magnetic Resonance Imaging
MNI: Montreal Neurological Institute
MRI: Magnetic Resonance Imaging
PET: Positron emission tomography
PTSD: Post-traumatic stress disorder
3D: Three-dimensional
